# 无固定相分离-电感耦合等离子体质谱法在环境中痕量金属纳米颗粒分析中的应用

**DOI:** 10.3724/SP.J.1123.2020.12016

**Published:** 2021-08-08

**Authors:** Haowen JIANG, Jian LI, Zhiqiang TAN, Yingying GUO, Yanwei LIU, Ligang HU, Yongguang YIN, Yong CAI, Guibin JIANG

**Affiliations:** 1.中国科学院生态环境研究中心, 北京 100085; 1. Research Center for Eco-Environmental Sciences, Chinese Academy of Sciences, Beijing 100085, China; 2.中国科学院大学, 北京 100049; 2. University of Chinese Academy of Sciences, Beijing 100049, China; 3.浙江环境监测工程有限公司, 浙江 杭州 310012; 3. Zhejiang Environmental Monitoring Engineering Limited Company, Hangzhou 310012, China; 4.国科大杭州高等研究院环境学院,浙江 杭州 310024; 4. School of Environment, Hangzhou Institute for Advanced Study, UCAS, Hangzhou 310024, China; 5.佛罗里达国际大学化学与生物化学系, 美国佛罗里达州 迈阿密 33199; 5. Department of Chemistry and Biochemistry, Florida International University, Miami 33199, United States

**Keywords:** 金属纳米颗粒, 流体动力色谱, 毛细管电泳, 场流分离, 电感耦合等离子体质谱, 单颗粒-电感耦合等离子体质谱, 综述, metal-containing nanoparticles (MCNs), hydrodynamic chromatography (HDC), capillary electrophoresis (CE), field-flow fractionation (FFF), inductively coupled plasma mass spectrometry (ICP-MS), single particle-inductively coupled plasma mass spectrometry (SP-ICP-MS), review

## Abstract

环境中金属纳米颗粒的分析检测不仅需要关注其浓度和化学组成,还需要对其形状、粒径和表面电荷等进行表征。此外,环境中金属纳米颗粒的分析需要解决其低赋存浓度以及复杂基质干扰的难题。无固定相分离技术与电感耦合等离子体质谱(ICP-MS)的在线联用,具有较强的颗粒分离能力和较低的元素检出限,能够快速准确地提供金属纳米颗粒的粒径分布、化学组成等信息,在金属纳米颗粒的分离检测方面表现出极大的潜能。但这一联用技术尚无法获得金属纳米颗粒物的颗粒数浓度和单个颗粒的元素信息,难以判断金属纳米颗粒涂层厚度、纯度以及颗粒的均相/异相团聚行为等。新兴的单颗粒-电感耦合等离子体质谱(SP-ICP-MS)与无固定相分离技术的在线联用,可以获得金属纳米颗粒的流体动力学粒径、元素质量计算粒径和颗粒数浓度等信息,进而弥补无固定相分离与ICP-MS在线联用技术的不足。该文介绍了流体动力色谱、毛细管电泳和场流分离3种常用无固定相分离技术的分离机制和适用检测器,着重综述了无固定相分离技术与ICP-MS/SP-ICP-MS在线联用技术的特点及其在环境金属纳米颗粒分析中的应用。关于场流分离,主要介绍了可以与ICP-MS联用的沉降场流分离和流场流分离。该文还对流体动力色谱、毛细管电泳和流场流分离与ICP-MS在线联用技术的特点进行了比较。最后,该文对无固定相分离技术与ICP-MS/SP-ICP-MS在线联用技术的发展提出了展望。

## Abstract (en)

Engineered metal-containing nanoparticles (MCNs), which have unique physical and chemical properties, are widely used in various fields such as medicine, pharmaceuticals, and microelectronics as well as in daily supplies. These MCNs are inevitably released into the environment during production and use, thus posing a threat to bacterial communities, animals, plants, and human health. There are also abundant natural MCNs in the environment, which play an important role in the environmental cycle of metals. The shape, size, and surface properties of MCNs have a significant impact on their migration, chemical and physical transformation, and biological intake in the environment. Therefore, the analysis and detection of MCNs in the environment should be aimed not only at quantifying their concentration and chemical composition, but also at determining their shape, particle size, and surface charge. In addition, for the detection of MCNs in the environment, challenges due to their low concentrations and the interference from complex environmental matrices must be overcome. A single detection technique is often insufficient for the analysis and detection of MCNs in a complex environment matrix. Therefore, the development of an effective and reliable online hyphenated technique is urgently needed for the separation and detection of MCNs in the environment. Such online hyphenated techniques should be able to eliminate the interference by complex matrices, improve the particle size detection range, and reduce the element detection limit. The online hyphenation of stationary phase-based separation techniques such as liquid chromatography and gel electrophoresis with inductively coupled plasma-mass spectrometry (ICP-MS) can effectively separate MCNs according to their particle size, with low element detection limits. However, these stationary phase-based separation techniques have a shortcoming of the adsorption of nanoparticles on the stationary phase, which leads to blockage of separation channels and low recoveries of nanoparticles. The online hyphenation of a non-stationary phase separation technique with ICP-MS also shows strong nanoparticle separation ability and low element detection limits, so that the problem of colloid blockage in stationary phase-based separation can be resolved. This method is very promising for the rapid and accurate characterization of the particle size distribution and chemical composition of MCNs. However, it cannot provide information about the nanoparticle number concentration of MCNs and the elemental content of a single MCN. In complex environmental samples, pure MCNs cannot be effectively distinguished from MCNs with environmental corona having different thicknesses or pure MCNs adsorbed on/hetero-agglomerated with inorganic/organic colloids. Online coupling single-particle ICP-MS (SP-ICP-MS), an emerging particle detection technique with non-stationary phase separation, can effectively help overcome the above shortcomings. This method can provide information on the hydrodynamic diameter, metal mass-derived diameter, total number concentration, size-dependent number, and size-dependent mass concentration of MCNs. Therefore, it enables comprehensive characterization of MCNs based on a variety of three-dimensional contour plot chromatograms. This review summarizes the separation mechanisms and applicable detectors for three commonly used non-stationary phase separation techniques: hydrodynamic chromatography (HDC), capillary electrophoresis (CE), and field-flow fractionation (FFF). In addition, it focuses on the characteristics and applications of online-coupling non-stationary phase separation with ICP-MS and SP-ICP-MS. Regarding FFF, this review focuses on the separation techniques that are suitable for online coupling with ICP-MS, such as sedimentation FFF and flow FFF (symmetrical flow FFF, asymmetrical flow FFF, and hollow fiber flow FFF). In addition, the characteristics of the online hyphenation of three non-stationary phase separations, HDC, CE, and flow FFF, with ICP-MS are compared, including the separation mechanism, sample volume, analytical time, detection sensitivity, size range, size resolution, recovery, reproducibility, and capability for ion analysis. Finally, this review proposes the prospects for future development of the online hyphenation of non-stationary phase separation techniques with ICP-MS and SP-ICP-MS.

金属纳米颗粒(MCN)是指含有金属的纳米颗粒,它可以是零价金属、金属氧化物、金属硫化物、多种金属复合或金属与自然胶体复合而成的纳米颗粒^[1]^。根据不同的来源,可将金属纳米颗粒划分为天然金属纳米颗粒和人工金属纳米颗粒。人工金属纳米颗粒是人为制造的一类金属纳米材料。由于独特的物理化学性能,人工金属纳米颗粒被广泛应用于医疗、制药、微电子、日常用品等各个领域^[2]^,其在制造和使用过程中不可避免地释放到环境中。例如,水冲刷可导致二氧化钛纳米颗粒(TiO_2_ NPs)从涂料与防晒霜中溶出^[3,4]^;含银衣物清洗可导致纳米银(AgNPs)溶出^[5]^,最终进入水体环境。天然金属纳米颗粒是指在自然条件下通过一系列氧化还原、络合、吸附/解吸等物理化学或微生物作用形成的金属纳米颗粒。例如,天然有机质可将金离子、银离子分别还原成纳米金(AuNPs)和AgNPs^[6,7]^;汞在溶解性有机质或细菌的作用下可以生成硫化汞纳米颗粒^[8]^。

人工与天然金属纳米颗粒均会与环境中的有机酸、无机盐、大分子聚合物等相互作用,导致其表面特性发生改变^[9]^。离子强度、光照、pH、阳离子类型、有机质和自然胶体的种类和浓度等也会影响金属纳米颗粒的分散和团聚(如均相团聚和异相团聚)^[9,10,11]^。金属纳米颗粒还可在自然条件下发生一系列化学转化,如氧化、氯化和硫化等^[11]^。这些金属纳米颗粒形貌结构、化学组成的变化使其原有的物理化学特性发生改变,从而进一步影响其环境迁移、转化与生物摄入^[12]^。

环境条件下金属纳米颗粒的分析检测是评估其环境迁移、转化、分布以及生物效应的前提。环境中金属纳米颗粒的分析检测不同于传统的溶解态金属。金属纳米颗粒的化学组成和粒径变化均会改变其原有的性质,因此不仅需要关注其化学组成和浓度,还需获得其形貌、粒径分布、纯度和表面电势等信息^[13,14]^。此外,分析检测金属纳米颗粒需要克服复杂环境基质的干扰及其低赋存浓度等难题。这给环境中金属纳米颗粒的分析检测带来了极大挑战^[15]^。

金属纳米颗粒常用的分析检测技术包括电镜、光散射、光学成像和单颗粒-电感耦合等离子体质谱(SP-ICP-MS)等。扫描电镜、透射电镜(TEM)可对纳米颗粒进行形貌、粒径和化学组分分析,但其对纳米颗粒含量要求较高(通常>1 mg/L),且样品制备过程可能导致颗粒团聚与形貌的改变^[16,17]^。动态光散射(DLS)、多角度光散射(MALS)和纳米颗粒跟踪分析(NTA)缺乏对颗粒的特异性识别,不能有效区分不同组分的颗粒,且多分散体系分析时更倾向于呈现大颗粒信息^[18,19,20]^。SP-ICP-MS作为一种新兴的纳米颗粒分析技术可识别不同的颗粒,提供粒径分布、颗粒浓度和离子浓度等信息,但无法实现小粒径金属纳米颗粒的检测,且相应离子浓度较高时会明显干扰小颗粒粒径的分析(例如10~80 nm)^[21]^。因此,单一检测技术往往无法实现复杂环境基质中金属纳米颗粒的分析检测。发展可靠的分离检测联用技术,去除复杂基质的干扰,提高粒径检测范围和灵敏度,是环境中金属纳米颗粒分析的关键。液相色谱(LC)、凝胶电泳(GE)等含固定相分离技术与ICP-MS的联用可有效对金属纳米颗粒按粒径进行分离,并具有较低的元素检出限^[22,23]^。但这类技术存在固定相对纳米颗粒的吸附问题,导致分离通道堵塞与纳米颗粒回收率低^[1]^。无固定相分离技术可以有效降低或避免固定相对纳米颗粒的吸附,提高金属纳米颗粒的回收率。高效无固定相分离技术与高灵敏ICP-MS/SP-ICP-MS的在线联用成为环境中金属纳米颗粒检测的有力工具。本文针对环境中金属纳米颗粒的分离检测,综述了目前常用的无固定相分离技术,如流体动力色谱(HDC)、毛细管电泳(CE)、场流分离(FFF)等与ICP-MS以及SP-ICP-MS的联用,并对相应技术的原理、应用和特点进行了总结与比较。

## 1 无固定相分离与传统ICP-MS在线联用技术

### 1.1 流体动力色谱

1.1.1 HDC-ICP-MS联用技术简介

在HDC色谱柱中,不同粒径纳米颗粒所受的水动力效应不同,大颗粒更易远离填充颗粒表面的低流速区域,而小颗粒更倾向于贴近填充颗粒移动。基于不同颗粒的速度梯度,HDC可以有效将金属纳米颗粒分离^[24]^。利用这一分离机制,HDC可使用无孔刚性颗粒填充色谱柱或使用不同孔径的空毛细管作为色谱柱。其中,无固定相HDC可有效避免尺寸排阻色谱等存在的固定相对金属纳米颗粒物吸附的问题^[25,26]^。HDC可以与不同的检测器同时联用,如DLS、MALS^[27]^、紫外-可见光谱(UV-Vis)^[28]^和ICP-MS^[29,30]^。DLS、MALS和UV-Vis作为ICP-MS检测的辅助手段可提供金属组成与浓度以外的辅助信息,如金属纳米颗粒的水合粒径、特征紫外可见吸收等。HDC-ICP-MS联用技术具有极强的稳定性,适用于基质复杂的环境样品,无需复杂的样品前处理^[29]^,不受待测颗粒密度、类型和环境温度的影响,且颗粒表面涂层对其保留行为影响也较小,因此无需特异性尺寸校准颗粒即可精确给出待测金属纳米颗粒物的粒径信息^[31]^。HDC-ICP-MS具有较宽的粒径分析范围(20~1200 nm)和较低的检出限(μg/L)^[29]^。此外,HDC-ICP-MS进行样品分析的时间较短(一般小于10 min)^[29]^。HDC-ICP-MS也存在一定的缺陷,如对复杂多分散颗粒物分离效果较差,但仪器操作条件的优化可以一定程度上缓解这一问题^[32]^。

1.1.2 HDC-ICP-MS联用技术应用

表1总结了HDC-ICP-MS在线联用技术在金属纳米颗粒分离检测中的应用。HDC-ICP-MS以去离子水或表面活性剂为载液,可以实现自然水体^[31,33]^、市政废水^[29,30,33]^、土壤^[34]^等环境基质中金属纳米颗粒的分离与检测,回收率可达60%~98%。采用去离子水为载液,可成功实现自然水体和市政废水中AgNPs的分离表征,如加标市政废水的回收率也可达到60%~70%^[33]^。但以去离子水为载液时,在分离过程中可能存在纳米颗粒团聚的问题,因此通常向载液中加入一定量的表面活性剂,以更好地分散金属纳米颗粒。同时,可向载液中加入缓冲液及甲醛(抗菌剂)^[29,35]^,以进一步提升分离的稳定性。以0.002 mol/L Na_2_HPO_4_、0.05%十二烷基硫酸钠(SDS)、0.2%非离子表面活性剂和0.2%甲醛作载液,在无需复杂样品前处理条件下即可实现污泥上清液中AgNPs、TiO_2_ NPs、SiO_2_ NPs、Al_2_O_3_ NPs和Fe_2_O_3_ NPs的分离表征,有效避免了金属纳米颗粒在样品前处理和分析过程中发生形貌变化,且单个样品分析时间一般小于10 min^[29-31,33]^。HDC-ICP-MS在固相样品中金属纳米颗粒的分离分析也具有极佳的性能。选用合适的提取剂可保持金属纳米颗粒物的原始状态,实现土壤和多种生活用品(如防晒霜)中金属纳米颗粒的快速粒径分离和元素组成分析^[34,35]^。在使用0.1% Triton X-100对土壤样品进行提取后,以0.5 g/L SDS、1 g/L Triton X-100、1 g/L Brij L23为载液实现了土壤提取物中含钛颗粒物的分离表征^[34]^。结合X射线荧光光谱(XRFA)、电感耦合等离子体发射光谱(ICP-OES)以及电镜分析,证明了土壤中含钛颗粒物主要以TiO_2_ NPs的形式存在^[34]^。在最优的实验条件下,HDC-ICP-MS可以实现对防晒霜中TiO_2_ NPs和ZnO NPs的分离定量,且回收率高达90%~98%^[35]^。

**表1:** HDC-ICP-MS在金属纳米颗粒分离检测中的应用

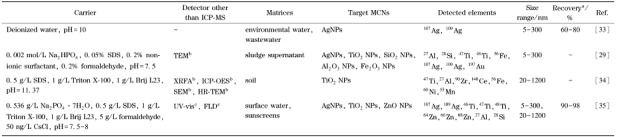

HDC-ICP-MS: hydrodynamic chromatography coupled with inductively coupled plasma mass spectrometry; MCN: metal-containing nanoparticle; a: calculated by ICP-MS; b: offline detector; C: online detector; SDS: sodium dodecyl sulfate; -: not mentioned; XRFA: X-ray fluorescence analysis; ICP-OES: inductively coupled plasma optical emission spectrometry.

### 1.2 毛细管电泳

1.2.1 CE-ICP-MS联用技术简介

CE采用弹性石英毛细管作为分离通道,在分离通道两端添加高压直流电场,使带电的纳米颗粒随电渗流向其电荷相反的方向迁移,根据不同的荷质比即可实现金属纳米颗粒的电泳分离^[36]^。CE在极小进样体积下可以有效分离不同粒径的固有荷电或吸附后荷电的金属纳米颗粒^[37]^。金属纳米颗粒的电泳淌度取决于其Zeta电位,而Zeta电位受颗粒的原有电荷状态、背景电解质等因素影响。因此,背景电解质溶液的改变、金属纳米颗粒对配体与表面活性剂的物理/化学吸附均会改变金属纳米颗粒的Zeta电位,进而影响CE对金属纳米颗粒基于尺寸的分离^[38,39]^。可与CE连接的检测器种类较多,LC适用的检测器理论上均可应用于CE的检测。但CE样品注射量仅为纳升,对环境样品中痕量金属纳米颗粒的分离检测往往需要与高灵敏度的ICP-MS联用^[37]^。

1.2.2 CE-ICP-MS联用技术应用

CE-ICP-MS是一种基于荷质比差异的分离技术。当不同粒径金属纳米颗粒本身荷质比差异较小时,CE-ICP-MS往往无法实现有效的分离测定。在这种情况下,通常需要添加表面活性剂来改变金属纳米颗粒的表面特性。例如,向分离电解质中加入离子型表面活性剂SDS时,颗粒表面的电势会发生改变;由于不同粒径金属纳米颗粒具有不同的表面积,因此对SDS的吸附能力存在差异,导致吸附SDS的金属纳米颗粒的电泳淌度正比于粒径;采用这一方式可实现不同粒径金属纳米颗粒的分离与表征^[40]^。分离电解质中表面活性剂的加入也有助于维持金属纳米颗粒的稳定性,抑制其团聚。此外,分离电解质中水溶性金属离子络合剂的使用可以抑制金属纳米颗粒对金属离子的吸附,且在特定浓度范围内的水溶性离子络合剂不会造成金属纳米颗粒的溶解,使金属纳米颗粒和金属离子的同时定量分析成为可能^[40,41,42]^。采用10 mmol/L 2-环己氨基乙磺酸、1 mmol/L硫普罗宁(银离子络合剂)和30 mmol/L Triton X-100作分离电解质,可实现6种膳食补充剂中AgNPs和银离子的分离检测^[42]^。CE-ICP-MS也是研究金属纳米颗粒转化的极佳工具。采用Na_2_HPO_4_ (pH 9.0)和Na_2_B_4_O_7_-H_3_BO_3_ (pH 9.0)分别作为分离电解质和样品电解质,可实现CdTe量子点及其降解产物Cd^2+^和$TeO_{3}^{2-}$的分离分析^[43]^。进一步地,CE-ICP-MS可以用于不同类型的量子点(这些量子点在核/壳化学组成、功能化和大小上都是可变的)在生物体内迁移转化的研究^[43,44,45]^。金属纳米颗粒与不同蛋白质的结合会影响其表面荷电状态,CE-ICP-MS可以根据不同的迁移时间有效区分金属纳米颗粒和金属纳米颗粒-蛋白结合物。此外,通过选择合适的分离电解质,实现与生理条件的兼容,CE-ICP-MS可以实现接近生理条件下金属纳米颗粒及相应蛋白结合物的分离表征^[46]^。采用40 mmol/L 4-羟乙基哌嗪乙磺酸(HEPES)作为分离电解质,CE-ICP-MS实现了纳米金、纳米金-白蛋白和纳米金-转铁蛋白的分离检测,且具有较高的回收率(86%~97%)和稳定性(迁移时间和峰面积精密度为1.0%~6.4%和2.4%~6.9%)^[46]^。CE-ICP-MS对金属纳米颗粒及相应蛋白结合物的分离表征也使其成为研究金属纳米颗粒物与蛋白分子化学反应(包括动力学和化学平衡分析)的可行工具,可用于计算反应速率常数、蛋白分子结合化学计量数^[46,47,48]^。纳米颗粒与蛋白质的结合也使得通过对纳米颗粒的高灵敏度分析实现蛋白质的间接定量成为可能。例如,已知纳米金与尿蛋白结合常数,可以向未知浓度的尿蛋白中添加过量AuNPs,通过CE-ICP-MS对过量AuNPs和AuNPs-尿蛋白结合物的分离检测即可实现尿蛋白的高灵敏定量分析^[49]^。表2总结了CE-ICP-MS在线联用技术在金属纳米颗粒分离检测中的应用。

**表2:** CE-ICP-MS在金属纳米颗粒分离检测中的应用

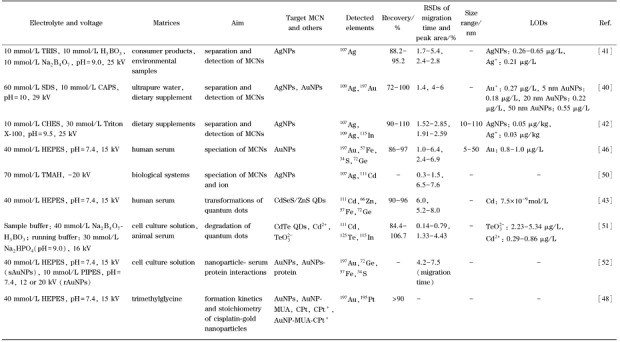

CE: capillary electrophoresis; TRIS: tris( hydroxymethyl) aminomethane; CAPS: N-cyclohexyl-3-aminopropanesulfonic acid; CHES : cyclohexylaminoethane sulfonic acid; HEPES: 4-(2-hydroxyethyl) piperazine- 1-ethanesulfonic acid; TMAH: tetramethyl-ammoniumhydroxide; sAuNPs: spherical AuNPs; rAuNPs: rod-shaped AuNPs; PIPES: piperazine-N,N'-bis( 2-ethane-sulfonic acid) ; QDs: quantum dots; MUA: 11-mercaptoundecanoic acid; CPt: cis-diamminedichloridoplatinum(II); CPt^*^ : active derivative of cisplatin; AuNP-MUA-CPt^*^ : conjugate of 11-mercaptoundecanoic acid modified AuNP and the active derivative of cisplatin.

### 1.3 场流分离

1.3.1 FFF-ICP-MS联用技术简介

场流分离是由Giddings课题组^[53]^在1966年提出的可用于大分子、胶体和颗粒分离的一种分离技术。当待测样品在载流承载下流经超薄分离通道时,在垂直于样品流动的方向施加一个可控的外加场,在外加场力和扩散力的共同作用下,使具有不同理化性质(粒径、密度等)的分析物在分离通道的一定距离达到平衡^[54]^。根据零滑移假设,距离分离通道壁近的载液流速小,而远的载液流速大,从而使处于不同流速载液中的分析物实现分离^[54]^。根据外加场力的不同,可将场流分离分为流场流分离(flow field-flow fractionation, FlFFF)、沉降场流分离(sedimentation field-flow fractionation, SdFFF)、热场流分离(thermal field-flow fractionation)和电场流分离(electrical field-flow fractionation)等^[55]^。目前使用较多的是FlFFF和SdFFF。

FlFFF和SdFFF均为不含固定相的分离技术,可有效避免分析物与分离通道相互作用而导致的损失^[56]^。SdFFF的外加力场是垂直于样品流动方向的离心力或重力,颗粒的分离取决于其当量球体直径和颗粒密度^[57]^。FlFFF的外加力场是垂直于样品流动方向的横向流,颗粒的分离取决于其流体动力学直径^[57]^。SdFFF具有较强的粒径分离能力,可以同时检测相应离子,但不适于小粒径颗粒(<50 nm)的分离,而且计算颗粒的粒径需要提前获知其颗粒密度^[58,59]^。相对于SdFFF, FlFFF理论更为成熟,分离条件更加温和,在分离过程中也不会破坏颗粒的形状,并具有更低的粒径检出限(1 nm)^[56]^。FlFFF主要包括对称流场流(symmetrical FlFFF, SF4)、非对称流场流(asymmetrical FlFFF, AF4)和中空纤维流场流(hollow fiber FlFFF, HF5)^[60]^。SF4和AF4均使用大体积的长方体平板膜作分离通道。但相对于SF4, AF4不仅具有交叉流作用下的样品松弛过程,还具有样品的聚焦过程,聚焦过程的存在使AF4具有更高的分离效率^[61]^。AF4还具有操作更简单灵活的特点,因此AF4的应用范围远远超过SF4。HF5采用小体积的中空纤维作分离通道,对样品的稀释小,所需载液体积少,分析时间短^[60]^。FFF-ICP-MS的分离分析性能受离子强度、pH、表面活性剂、聚焦时间、流速和外加场力等因素影响。在场流分离过程中,应尽可能保持金属纳米颗粒在分析过程中的稳定性和分散性,减少膜对金属纳米颗粒的吸附,确保获得最佳的分离效果^[62,63,64]^。

1.3.2 FFF-ICP-MS联用技术应用

SdFFF是最早与ICP-MS联用的场流分离技术。SdFFF-ICP-MS具有较强的粒径分离能力,但粒径检出限较高,且外加场易导致颗粒物形状的改变,因此目前主要应用于不易发生形状改变的金属纳米颗粒及环境胶体的分离表征和迁移转化研究中^[56,65-67]^。在使用己烷去除化妆品中有机物之后,采用SdFFF-ICP-MS可以实现化妆品中TiO_2_ NPs的分离表征^[65]^。利用SdFFF-ICP-MS可对不同粒径环境胶体进行分析,可获得这些环境胶体的元素组成(常量、微量和部分痕量元素)。SdFFF-ICP-MS揭示,表层土壤中铁主要富集于小粒径与大粒径胶体中,而非中等粒径胶体(~0.3 μm)^[66]^。SdFFF-ICP-MS也被用于分析不同粒径胶体的元素比(如Fe/Al、Mg/Al),并进一步推断胶体的矿物组成^[66,67]^。元素比分析显示,高岭土、蛭石、伊利石矿物在较宽的土壤粒径范围内均存在;相对而言,高岭土在较大尺寸胶体中(>0.3 μm)较少^[67]^。利用土壤胶体粒径和元素组成信息,可以推测土壤胶体导致的金属运移^[66]^。

FlFFF-ICP-MS温和的分离方式、强大的颗粒分离能力和较低的检出限使其广泛用于日常消费品、复杂环境介质和生物组织中金属纳米颗粒的分离表征。借助高效提取剂,FlFFF-ICP-MS可应用于水体、土壤和沉积物中金属纳米颗粒的分离表征。例如,以Na_4_P_2_O_7_为提取剂,采用AF4-ICP-MS成功实现了表层水中AgNPs、CeO_2_ NPs和Fe_2_O_3_ NPs等纳米颗粒的分离表征^[68]^;类似地,以Na_4_P_2_O_7_为提取剂,采用AF4-HR-ICP-MS可实现土壤中含铁氧化物颗粒的粒径分布和元素组成分析^[69]^。选择合适的有机溶剂作载液还可以实现非水溶性样品中金属纳米颗粒的分离表征。以四氢呋喃为载液,AF4-UV-MALS-ICP-MS/MS联用实现了石油烃气体冷凝物中金属纳米颗粒(如含汞纳米颗粒)的分析,金属颗粒的回收率为75%左右,相关结果得到了扫描透射电镜-能量色散X射线光谱(STEM-EDX)的验证^[70]^。FlFFF-ICP-MS还可有效区分金属纳米颗粒及金属纳米颗粒-蛋白结合物。例如,使用SF4-ICP-MS研究AgNPs与牛血清白蛋白、球蛋白和纤维蛋白相互作用,可得到AgNPs与这些蛋白质的结合常数^[71]^。另外,采用AF4-ICP-MS可以识别CdSe/ZnS核/壳量子点与单克隆IgG抗体的偶联产物,量化量子点与抗体的生物偶联效率^[72]^。

金属纳米颗粒在环境中的形态转化对于理解其环境与生物效应具有重要意义。FlFFF-ICP-MS的高粒径分辨率和高检测灵敏度使其可以有效分离和识别新生成的纳米颗粒,进而研究环境中颗粒物的演变,实现对新生成颗粒物的识别和定量。如AF4-MALS-ICP-MS能够精准识别AgNPs在光照下的粒径变化,进而研究AgNPs在光照下的溶解与团聚行为^[73]^。DLS对纳米颗粒的环境团聚研究通常需要采用较高的浓度(~1 mg/L),其结果难以推广至实际环境中低浓度人工纳米颗粒的团聚行为。Tan等^[74]^基于HF5-ICP-MS优异的粒径分离能力和极低的检出限,实现了10 μg/L AgNPs在复杂环境基质(溶解性有机质、Na^+^/Ca^2+^)中的均相团聚研究,为环境相关浓度下人工金属纳米颗粒的团聚研究提供了新的手段。由于半透膜的存在,FlFFF聚焦与分离过程中往往会造成离子和小粒径纳米颗粒透出,因而无法实现离子、小粒径与大粒径颗粒的同时分析。通过在HF5径向流出口位置连接富集小柱(MCC)实现银离子的捕集,然后通过硫代硫酸钠对MCC进行洗脱可以实现银离子的定量分析;小粒径(如小于2 nm)AgNPs和强络合态银离子无法被MCC捕集而直接进入检测器;在MCC和HF5中间连接位置额外添加2% H_2_O_2_可使90%以上的小粒径AgNPs氧化,仅强络合态银离子直接进入检测器,通过两次检测的差值即可实现小粒径AgNPs的定量^[75]^。采用以上手段,利用HF5-MCC-ICP-MS可成功实现银离子和不同粒径AgNPs的同时分离表征^[75]^。HF5-MCC-ICP-MS对多种银形态的定量分析优势也使其分析环境相关浓度(10~33.97 μg/L)下银离子和AgNPs的转化成为可能。研究发现,环境相关浓度下银离子在光照下可生成小粒径AgNPs(<2 nm与2.3 nm),而银离子和AgNPs在废水中会部分转化为巯基结合态一价银^[74]^。HF5-MCC-ICP-MS有效弥补了UV-Vis、DLS、TEM等常规分析手段对环境浓度下金属纳米颗粒表征的缺陷以及AF4-ICP-MS对离子与小粒径金属纳米颗粒分析的不足^[74,75]^。表3对SdFFF-ICP-MS、HF5-ICP-MS、SF4-ICP-MS和AF4-ICP-MS在金属纳米颗粒分离方面的应用进行了列举。

**表3:** FFF-ICP-MS在金属纳米颗粒分离检测中的应用

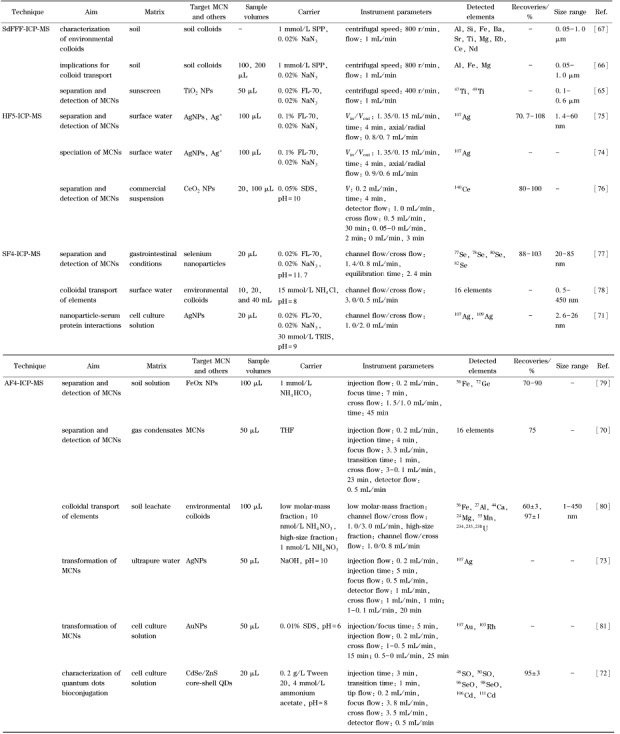

FFF: field-flow fractionation; SdFFF: sedimentation field-flow fractionation; HF5: hollow fiber flow field-flow fractionation; SF4: symmetrical flow field-flow fractionation; AF4: asym-metrical flow field-flow fractionation; SPP: sodium pyrophosphate; THF: tetrahydrofuran.

## 2 无固定相分离与SP-ICP-MS在线联用技术

### 2.1 无固定相分离-SP-ICP-MS简介

流体动力学粒径、单个颗粒金属质量计算粒径等综合信息有助于判断金属纳米颗粒涂层厚度、纯度以及颗粒的均相/异相团聚行为。SP-ICP-MS作为一项新兴的金属颗粒检测技术,能够对金属纳米颗粒的质量计算粒径、粒径分布和颗粒浓度进行表征,且具有极低的颗粒数检出限^[82]^。HDC、CE和FFF等无固定相分离与SP-ICP-MS在线联用技术可在一次分析中获得金属纳米颗粒流体动力学粒径、元素质量计算粒径、颗粒物浓度等信息,三维图像的绘制使金属纳米颗粒的信息更加直观化,弥补了单一技术分析的不足^[83,84,85]^。

### 2.2 无固定相分离-SP-ICP-MS应用

SP-ICP-MS低颗粒数检出限的优势使得HDC-SP-ICP-MS可以实现饮用水中低至ng/L水平AuNPs的分析,流体动力学粒径、单颗粒质量计算粒径和颗粒浓度的获取使其对AuNPs的表征更为全面^[86]^。HDC可以分离金属离子和相应纳米颗粒,因此HDC的预分离过程不仅可实现金属纳米颗粒和溶解态离子的同时表征,还有效避免了SP-ICP-MS金属纳米颗粒分析中金属离子的干扰,降低了金属纳米颗粒的粒径检出限^[87]^。一般而言,球状金属纳米颗粒的流体动力学粒径与单颗粒质量计算粒径相近,而棒状金属纳米颗粒的流体动力学粒径大于质量计算粒径^[31]^。因此,对于较纯的球状与棒状金属纳米颗粒,通过HDC-SP-ICP-MS获得的元素质量计算粒径和流体动力学粒径的对照即可区分球状和棒状金属纳米颗粒^[31]^。类似地,均相聚集体的流体动力学粒径与质量计算粒径相近,而异相聚集体的流体动力学粒径远大于其质量计算粒径,因此HDC-SP-ICP-MS也可区分金属纳米颗粒的均相聚集体和异相聚集体^[83]^。

CE的进样体积仅在nL范围。因此,在较小的进样体积下,当分析物浓度较低时,需要考虑分析过程获得的金属纳米颗粒的颗粒数是否具有统计学意义。在这一背景下,采用在线预浓缩手段,提高颗粒物浓度显得尤为重要。使用反电极极性堆积模式对AgNPs进行预浓缩,可以使CE-SP-ICP-MS的灵敏度提高14.3~27.7倍^[88]^。具有不同涂层的金属纳米颗粒在CE中具有不同的电泳淌度,但当其电泳淌度差异较小时往往无法被完全分离^[38]^。CE-SP-ICP-MS可以获得具有相同迁移时间颗粒的质量计算粒径信息,进而实现了20、40、60 nm柠檬酸包裹AgNPs和40、60 nm聚乙烯吡咯烷酮包裹AgNPs混合物的分离表征^[85]^。

虽然通常情况下,FlFFF-SP-ICP-MS无法实现溶解态离子的分析,但FlFFF聚焦与分离过程金属离子的透析去除减少了离子背景对SP-ICP-MS金属纳米颗粒分析的影响,降低了SP-ICP-MS的粒径检出限^[84,89]^。通过对银离子的去除,AF4-SP-ICP-MS可实现低至ng/L水平AgNPs的分离表征;通过监测AgNPs在迁移过程中的粒径变化并结合扫描透射电镜和能量色散X射线光谱,证明了AgNPs迁移过程中均相聚集体和银/聚合物异相聚集体的形成^[89]^。与其他基于SP-ICP-MS的联用技术类似,AF4-SP-ICP-MS可以获取金属纳米颗粒的多维信息,通过三维图像的建立可以有效区分具有相同银质量的AgNPs和Ag-SiO_2_核/壳纳米颗粒^[84]^。表4对HDC、CE、FFF与SP-ICP-MS在线联用技术在金属纳米颗粒分离表征方面的应用进行了总结。

**表4:** 无固定相分离与 SP-ICP-MS在线联用技术在金属纳米颗粒分离检测中的应用

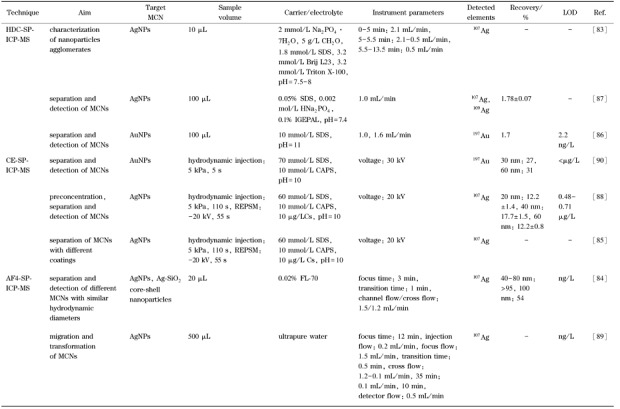

SP-ICP-MS: single particle- inductively coupled plasma mass spectrometry; REPSM: reversed electrode polarity stacking mode; IGEPAL: IGEPALB CA-630 nonionic detergent.

## 3 无固定相分离技术的比较

HDC、CE和FFF与ICP-MS联用技术在对环境中金属纳米颗粒的分离检测方面表现出了极佳的能力,但3种分离技术也有自身的优势和不足。FlFFF-ICP-MS为FFF-ICP-MS中理论更为成熟、应用更为广泛的在线联用技术。表5对HDC-ICP-MS、CE-ICP-MS和FlFFF-ICP-MS的特点进行了比较。在进行环境中金属纳米颗粒的分离检测时应根据具体的研究问题选择合适的无固定相分离技术。

**表5:** 3种无固定相分离技术与ICP-MS在线联用技术相关特点总结

Method	Separation mechanism		Analyte		Sample volume		Analytical time/min		Detection sensitivity		Size range		Size resolution		Recovery		Reproduc- ibility		Capability for ion analysis	
HDC-ICP- MS		hydrodynamic effect		no limit		20-100 μL		<10		low		5-1200 nm		low		high		high		direct analysis
CE-ICP-MS		charge to size ratio		self-charged or chargeable substances		1-10 nL		10-15		low		0.1-2000 nm		high		high		low		direct analysis
FlFFF-ICP- MS		diffusion coef- ficient		no limit		1 μL- 50 mL		20-30		high		1 nm-100 μm		medium		low		high		indirect analysis

HDC、CE和FlFFF是分别基于水动力效应、电泳淌度和扩散系数3种不同分离机制的颗粒分离技术^[58]^。由于分离机制的不同,不同分离技术对待测物的要求也存在差异。HDC和FlFFF对分析物没有特殊要求,而CE则需要待测颗粒物具有不同的荷质比,其可以通过本身荷电或化学修饰荷电,进而获得不同荷质比。HDC相较于其他两种分离技术具有更强的抗干扰能力和适应性,颗粒保留系数受颗粒涂层、颗粒密度、温度和流速变化的影响较小或不受影响,因此某一颗粒制成的尺寸校准曲线可用于其他不同类型的颗粒^[31,35]^。CE对金属纳米颗粒的分离基于颗粒的电泳淌度,受颗粒电荷状态、背景电解质的影响,因此电荷状态、背景电解质、电渗流和温度的变化以及颗粒与毛细管壁的相互作用均会影响分离的可重复性,选择合适的分离介质条件和粒径校正标准才能获得未知样品的可靠粒径信息^[38,39,91]^。颗粒物在FlFFF中的保留时间受载流流速、外加场力、通道体积、通道高度、颗粒物尺寸以及膜对颗粒吸附的影响,在使用FlFFF对环境中金属纳米颗粒进行分析时要选择合适的离子强度、pH、表面活性剂、聚焦时间、载流流速和外加场力等,保证金属纳米颗粒在分析过程的稳定性和分散性,减少膜对金属纳米颗粒的吸附^[62,63,64]^。

HDC、CE和FlFFF在粒径分离范围方面存在一定的差异。FlFFF(1 nm~100 μm)具有更大的粒径分离范围,适用于其他技术无法分离的大粒径颗粒物的分离分析。HDC(5~1200 nm)和CE(0.1~2000 nm)分离粒径相近,但HDC对小粒径颗粒物的分离效果较差^[32,37]^。HDC、CE和FlFFF在进样量方面存在明显差异。受限于色谱柱和毛细管的体积,HDC和CE为了避免谱峰加宽,样品进样量分别为20~100 μL和1~10 nL,仅为分离装置体积的10%左右。而FlFFF在样品分离之前存在聚焦过程,进入装置的颗粒物会在样品输入端附近聚焦,因此大体积的样品可以直接注入FlFFF系统中,而不会使谱峰加宽,样品的进样体积可以从几微升到几十毫升^[37,58,60]^。大体积进样的特点使FlFFF对环境中金属纳米颗粒的检测灵敏度明显高于HDC和CE,使其更加适用于环境中低浓度金属纳米颗粒的分离检测^[37]^。

此外,HDC、CE和FlFFF在分离时间、尺寸分辨率和样品回收率方面存在一定的差异。聚焦过程和外加场力的存在使FlFFF相对于HDC具有较好的粒径分离效果,但同样增加了颗粒的分离时间。

FlFFF系统中半透膜对颗粒物的吸附导致其样品回收率较低^[1,58,60]^。HDC操作简单易于实施,分析时间仅为10 min左右,拥有较好的样品回收率,但存在粒径分辨率较低的问题^[32]^。相对于其他分离技术,CE具有更高的粒径分辨率,同时颗粒回收率较高^[58]^。HDC和CE可在一次实验运行中同时对离子态和颗粒态金属进行分析^[1]^。FlFFF由于聚焦过程中金属离子可透过半透膜,通常FlFFF不能直接对溶解态离子进行分析。但FlFFF分析中,通过在线微柱富集透析出的金属离子,经过洗脱也可对溶解态离子进行分离定量^[74,75]^。

## 4 结论与展望

环境中人工与天然金属纳米颗粒的表征与定量对分析方法提出了挑战。无固定相分离技术与ICP-MS的在线联用可以提供金属纳米颗粒的粒径分布、化学组成、颗粒浓度等信息,在环境金属纳米颗粒的分离检测中表现出极大的潜力。

但在实际应用过程中,无固定相分离与ICP-MS在线联用技术仍存在一定的缺陷和不足,虽然其具有较低的元素检出限,但环境中一些金属纳米颗粒的含量极低,实际环境浓度下金属纳米颗粒的迁移转化研究仍受到一定程度的制约。颗粒物的形状对其在无固定相分离装置中的保留时间有重要影响,而无固定相分离技术的校正曲线多基于待测颗粒物为均一密度的球体,对实际环境样品中非球体金属纳米颗粒的分析会存在偏差。纳米颗粒标准物质的认证值通常为粒径大小,浓度在某些情况下仅具有指示作用,缺乏基于纳米颗粒物尺寸和颗粒数浓度认证的标准物质来进行浓度校准。因此,在未来应进一步研究非球体颗粒物在无固定相装置中分离行为及其校正方法,同时发展基于颗粒物形状、颗粒物尺寸和颗粒数浓度认证的标准物质,以应对实际环境金属纳米颗粒分离检测的需求。

无固定分离技术与ICP-MS联用可以充分发挥分离技术对颗粒物的粒径分离以及ICP-MS的高灵敏度和多元素分析的优势,进而获得颗粒的粒径分布和化学组成,但其难以区分不同涂层的金属纳米颗粒,无法对单个颗粒的多元素化学组成进行分析。无固定相分离技术与SP-ICP-MS的联用可以通过比较颗粒物流体动力学粒径和质量计算粒径的差异对颗粒的纯度进行分析,进而区分均相聚集体和异相聚集体。但SP-ICP-MS只能对单元素进行分析而无法获得单个颗粒的多元素化学组成。近年来发展的SP-ICP-TOF-MS可以对单个纳米颗粒上的多种元素进行同时分析,有望根据特定元素组成识别人工与天然金属纳米颗粒。无固定相分离技术与SP-ICP-TOF-MS的联用将会为环境中金属纳米颗粒的分析检测提供新的信息,促进金属纳米颗粒的环境过程、地球化学循环、生态毒理学等相关研究的深入开展。
